# Predator Size Structure Fails to Alter Nonconsumptive Effects in Streams

**DOI:** 10.1002/ece3.72539

**Published:** 2025-11-24

**Authors:** Benjamin J. Toscano, Alyce Segal, Martina Exnerova, Mia A. Ver Pault

**Affiliations:** ^1^ Department of Biology Trinity College Hartford Connecticut USA

**Keywords:** fear, functional trait, intraspecific, nonlethal, ontogeny

## Abstract

Predator population size structure varies over space and time, mediating the top‐down, consumptive effects of predators on ecosystems. Yet the role of predator size variation in governing nonconsumptive predator effects has received little targeted research attention. We manipulated stonefly ( *Acroneuria abnormis* ) predator size structure and feeding ability and measured effects on the benthic invertebrate prey community in a headwater stream. Field enclosures retained stonefly predators but allowed smaller prey to emigrate as a behavioral avoidance response. Stoneflies caused a ~30% reduction in total prey abundance regardless of whether or not they could feed, indicating a major role for nonconsumptive effects in determining the overall predator effect. This pattern was consistent across two different stonefly predator size structures with equivalent total biomass, as well as most prey responses measured at both the community level and the individual taxon level. Our study demonstrates that stonefly predators cause a community‐scale nonconsumptive effect and suggests that predator biomass, rather than predator size structure, might determine the strength of this effect.

## Graphical abstract

Stonefly predators caused pervasive nonconsumptive effects on benthic invertebrates in a headwater stream. These effects did not depend on stonefly size structure. Predator biomass may be the key determinant of stonefly induced nonconsumptive effects in streams.

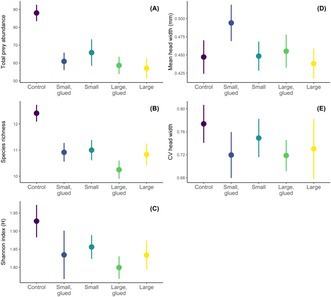

## 1 Introduction

Understanding how predators shape communities is a core ecological goal. While the killing and consumption of prey (consumptive effects—CEs) is a major influence on community structure, predators can additionally alter the behavioral, physiological, morphological or life history traits of prey in the absence of killing (Werner and Peacor 2003; Preisser et al. 2005). Many of these nonconsumptive effects (NCEs) occur downstream of prey behavioral changes that reduce the risk of predation, but also the energy available for prey growth and reproduction (Wirsing et al. 2021). Although the importance of NCEs as a community‐structuring force is established (Werner and Peacor 2003; Preisser et al. 2005), the factors that determine the strength of NCEs and their importance relative to CEs within and across ecological systems remain open areas of study (Wirsing et al. 2021; Orrick et al. 2024).

Body size is a dynamic property of individuals that underlies many predator–prey traits (Werner and Gilliam 1984; Miller and Rudolf 2011), and thus variation in population size structure may help explain NCE variability (Thaler and Griffin 2008; Hill and Weissburg 2013; Pessarrodona et al. 2019). For example, many prey reach a size refuge from predation, experiencing weaker CEs as they grow. NCE strength is expected to mirror this decline because anti‐predator behavior in the absence of risk is harmful (Helfman 1989). Yet instead, experimental studies instead show that large, less‐vulnerable prey individuals exhibit similar (Thaler and Griffin 2008) or stronger (Pessarrodona et al. 2019) anti‐predator behavioral responses than smaller, more vulnerable conspecifics, with cascading effects on prey resources (Pessarrodona et al. 2019).

Despite these insights into the effects of prey size variation, few studies have tested the effects of intraspecific size variation at higher, predator trophic levels on NCEs (but see Hill and Weissburg 2013). This paucity of data limits our ability to predict top‐down effects given natural variability in predator size structure and anthropogenic effects on predator size structure over time and across space (Gardner et al. 2011; van Wijk et al. 2013). CEs of predators are well known to depend on predator body size (Brose 2010; Miller and Rudolf 2011; Cozzoli et al. 2022). Larger predator individuals generally have greater *per capita* CEs than smaller conspecifics due to the need to satisfy greater metabolic demands and/or release from size‐related feeding constraints (e.g., gape size or mobility limitations) (Chalcraft and Resetarits 2004; Brose 2010). Thus, adaptive theory predicts that prey will exhibit greater behavioral adjustments in response to large predator individuals, that is, stronger NCEs (Helfman 1989).

However, evidence for “ontogenetic coupling” in CEs and NCEs (sensu Pessarrodona et al. 2019) is limited, and how prey gather information regarding predator size structure is currently unclear. For example, in aquatic systems, waterborne chemical cues produced by predators are a primary means of predator detection. In such systems, prey behavioral responses may depend simply on the concentration of chemical cues in a given location, which should increase with predator body size, but also predator density and the degree of predator spatial aggregation (Hill and Weissburg 2013). Under this mechanism, information on predator body size per se is not gathered—prey fail to distinguish between a harmless group of small predators versus a single, but dangerous large predator with the same total biomass (Hill and Weissburg 2013).

However, functional differences among predator size classes may allow prey to discriminate predator size structure as a unique characteristic apart from biomass. Many predator species change the types of prey they consume, their foraging tactics and space use as they grow (Werner and Gilliam 1984; Miller and Rudolf 2011). Such ontogenetic niche shifts change the nature of cues that predators emit, allowing prey to more accurately assess the risk posed by differently sized predators. For example, prey respond differently to conspecific predators fed different diets (Scherer and Smee 2016), but such occurs naturally within predator species that change their diets over ontogeny. Similarly, hunting mode differences across predator species have been shown to determine the strength of NCEs (Wirsing et al. 2021), but predators also exhibit changes in foraging tactics as they grow, determining the nature of tactile or visual cues that determine prey behavioral responses (Werner and Gilliam 1984). Thus, in systems with ontogenetic niche shifts, NCEs may depend on more than just predator biomass and its effects on cue intensity.

Our study manipulated the size structure of predatory stonefly nymphs ( *Acroneuria abnormis* ; Family Perlidae) in field enclosures and measured their NCEs and CEs on the benthic invertebrate community in a headwater stream. Perlid stoneflies are important mesopredators whose diet consists primarily of smaller aquatic insects (e.g., Ephemeroptera, Trichoptera, Diptera) but also plant matter and detritus (Tierno de Figueroa and López‐Rodríguez 2019). Some variation in reported stonefly diets (e.g., carnivory vs. omnivory) can be explained by stonefly instar body size; many Perlid species exhibit ontogenetic diet shifts, consuming an omnivorous diet in early instars before transitioning to feeding exclusively on insects in later (larger) instars (Sheldon 1969; Allan 1982; Duvall and Williams 2000). In later instars, stonefly CEs can reduce the abundance of particular insect prey taxa in streams (Peckarsky and Dodson 1980; Peckarsky 1991; Khamis et al. 2015), thereby modifying prey community structure. Though less studied, stoneflies can also affect insect prey via NCEs, causing behavioral changes that reduce prey food intake, growth, and fecundity (Peckarsky et al. 1993; McIntosh and Peckarsky 1999; Wellnitz 2014). These aspects, and the manipulability of stoneflies over small field scales, allowed us to experimentally address the question of how NCEs and their importance relative to CEs are influenced by predator body size variation.

Our field experiment used two stonefly predator size structure treatments (four small individuals vs. two large individuals) with equivalent total biomass. We crossed this factor with a mouthpart gluing manipulation that precluded stonefly feeding to isolate the effects of predator size structure on NCEs. These treatments were maintained in field enclosures that allowed for prey immigration and emigration, the latter a common NCE on insect prey in streams (Waters 1972; Malmqvist and Sjöström 1987).

Given the potential for stonefly ontogenetic diet shifts from omnivory to carnivory, we hypothesized that larger stoneflies would induce greater CEs on invertebrate prey, though other relationships between predator body size and CEs are possible. For example, smaller predators have a greater mass‐specific metabolic rate, and thus a group of small predator individuals should cause stronger CEs than a group of larger predator individuals with equivalent total biomass (Chalcraft and Resetarits 2004). Likewise, the effect of stonefly size structure on NCEs may depend on the degree of stonefly niche shifts. In the absence of niche shifts (or when prey fail to respond to concomitant cue changes), total predator biomass, rather than size structure per se should govern the strength of NCEs in aquatic systems (Hill and Weissburg 2013). In this case, we expect no change in NCEs with predator size because our two size structure treatments were equivalent in biomass. However, in niche‐shifting predators such as stoneflies, it is possible that large predator individuals produce qualitatively different cues that increase NCEs independent of biomass. In this case, NCEs should mirror CEs, establishing ontogenetic coupling in these effect types.

## 2 Methods

### 2.1 Study System

We conducted our study in riffle sections of Tankerhoosen Brook (41°49′45″ N, 72°27′35″ W) in Vernon, CT USA from June through July 2022. Tankerhoosen Brook is a cold‐water, spring‐fed stream with a watershed area of 33.2 km^2^ (Neumann and Wildman 2002). Our study section has an active channel width of 4–5 m, is partially shaded by deciduous forest, and contains a substrate composed of small to large rocks (25 cm diameter) over gravel and sand. The summer period over which our study was conducted is characterized by dryness and low flows and we timed experimental blocks to avoid rain events and runoff that would change water chemistry (Stewart et al. 2022) and dislodge enclosures.

We used *Acroneuria abnormis* as the predator in our study, the numerically dominant Perlid stonefly in Tankerhoosen Brook and a widely distributed species across the United States and Canada (Peckarsky 1979). Perlid stoneflies play important roles as mesopredators in the Tankerhoosen, consumed by wild brook trout ( *Salvelinus fontinalis* ) and brown trout ( *Salmo trutta* ) while consuming smaller aquatic insects. The insect prey community in the Tankerhoosen is dominated by dipterans (namely chironomids), mayflies, caddisflies, and other primarily detritivorous stonefly (Plecoptera) taxa.

### 2.2 Experimental Design

Our field experiment examined how stonefly size structure influenced the strength of NCEs relative to CEs on various aspects of prey community structure. We crossed stonefly size structure (small versus large) with a mouthpart gluing manipulation (unglued versus glued) that precluded CEs, yielding four unique treatments. We additionally conducted a no‐predator control. The experiment was run in a complete block design from June 21, 2022 to July 24, 2022 with six temporal blocks run in total.

We manipulated stonefly size structure with two levels—four small individuals (3.7–4.1 mm head capsule width, mean ± 1 SD = 3.93 mm ± 0.28) versus two large individuals (4.6–5.0 mm head width, mean ± 1 SD = 4.77 mm ± 0.20). These size structures are equivalent in total biomass based on an established length‐mass relationship for *Acroneuria abnormis* (Benke et al. 1999). We chose to hold stonefly biomass constant to isolate the effects of predator size structure; holding density constant would confound size structure with total biomass, the two factors we hypothesized would influence NCEs. These treatment levels further yielded stonefly densities on the high end of field estimates from Tankerhoosen Brook (personal observations).

We isolated NCEs from the total stonefly predator effect by gluing stonefly mouthparts. Glued stoneflies are only capable of altering the traits of prey (e.g., behavior), while unglued stoneflies can also consume prey (i.e., a combination of NCEs and CEs). We note, however, that the act of prey consumption and the concomitant release of prey alarm cues can also elicit prey behavioral responses, and gluing stonefly mouthparts removes this NCE pathway. We glued stonefly mouthparts by gently drying mouthparts with a paper towel and applying a small drop of Barge Cement with a probe (Peckarsky and Lamberti 2017). Previous studies using this manipulation demonstrate that mouthpart gluing precludes stonefly feeding, but does not affect stonefly foraging behavior (Peckarsky et al. 1993) or survival (Wellnitz 2014; Morton et al. 2022).

### 2.3 Experimental Setup

We manipulated these two factors, stonefly size and feeding ability, within in‐stream enclosures. Enclosures were plastic containers (15 cm × 10 cm × 8 cm) with the sides and tops cut out and replaced with 2‐mm mesh panels (Peckarsky and Lamberti 2017). We used 2‐mm mesh to retain larger stonefly predators, but allow for immigration and emigration of smaller prey species (Cooper et al. 1990; Sih and Wooster 1994). Thus, prey could respond behaviorally to stonefly predators by leaving an enclosure and emigrating to a new habitat patch (e.g., via drift) (Waters 1972; Malmqvist and Sjöström 1987). Two millimeter mesh further allows for more natural flow dynamics within enclosures and reduces sedimentation compared to smaller mesh sizes (Peckarsky and Lamberti 2017).

All experimental blocks were placed in the same riffle to minimize variation in environmental conditions including shading, current velocity, and substrate type. Each enclosure was stocked ~¾ full of 15 stones of similar size (~5 cm diameter) collected from riffles outside the experimental area to anchor enclosures in the substrate and provide natural habitat. Stone surfaces carried the benthic invertebrate community at natural abundances. Any Perlid stoneflies or other larger predators (e.g., Megaloptera, Odonata) that would alter the intended treatments were removed before placing stones into enclosures.

Stoneflies used in the experiment were collected using 500 μm rectangular kick nets from outside the experimental riffle. We identified stoneflies to the species level, measured head capsule width using electronic Vernier calipers (Mitutoyo, model CD‐6″ ASX) with an accuracy of ±0.01 mm, glued stonefly mouthparts and added stoneflies to enclosures. Unglued stoneflies were subjected to a “sham” procedure in which they were handled in the same manner as glued stoneflies before being placed into enclosures.

Enclosures were placed haphazardly with respect to treatment but roughly equally spaced ~1 m apart within the experimental riffle. Rectangular enclosures were placed with their long axes parallel to the stream flow direction to minimize drag and avoid dislodgement. We used a flow probe (Global Flow Probe FP101) to keep near‐bed current velocities as similar as possible among enclosures within each experimental block. Once a suitable flow location for an enclosure was determined, we pushed aside larger rocks from the area and buried enclosures ~4 cm into the substrate. Enclosures were further anchored by loosely piling rocks on all four sides and on top to prevent dislodgement (Peckarsky and Dodson 1980). Rocks did not prevent prey from entering or exiting enclosures.

The experiment was run for 72 h. This experimental duration was determined based on previous research showing that after longer time periods, sedimentation and fouling can alter environmental conditions within enclosures (Peckarsky et al. 1990). Both before and after each experimental block, we measured the near‐bed current velocity in front of each individual enclosure and the water temperature of the experimental riffle. Patch‐scale variation in near‐bed current velocity can influence top‐down effects of stoneflies (Peckarsky et al. 1990; Wellnitz 2014), whereas measuring water temperature allowed us to account for environmental change across temporal blocks.

We ran a total of six complete temporal blocks with two treatment replicates and two or four control replicates per block, resulting in 12 treatment replicates and 18 control replicates in total (*n* = 66 experimental units). This level of replication was based on previous studies measuring stonefly predator effects on benthic invertebrate communities in streams (Peckarsky and Dodson 1980; Peckarsky 1991). Due to the presence of a large stonefly at the end of 72 h, a single control replicate was dropped before the analysis. Thus 65 experimental units were used in our analysis.

### 2.4 Data Collection

After 72 h, each enclosure was removed from the stream with a kick net to capture any displaced insects. The contents of enclosures were placed in white enamel trays and all stonefly predators and prey were recovered, preserved in 70% ethanol, and brought back to the lab for identification and head capsule width measurements. Prey identification took place within the following days. All potential prey (*n* = 4406) were identified down to the lowest taxonomic resolution possible—to the genus or species level, except for Dipterans (family level), Plecoptera (order level), and caddis pupa (order level). We additionally measured the head capsule width of each insect prey individual when intact (*n* = 4153) under a dissecting scope (LAXCO, model LMS‐Z230P) equipped with an ocular micrometer. Prey head width measurements were used to examine whether stoneflies modified prey size structure, in addition to abundance‐based metrics of prey community structure.

### 2.5 Analysis

We used linear mixed models (LMMs) to assess the effects of experimental manipulations (stonefly presence, size structure and mouthpart gluing) and environmental variables (current velocity, stream temperature) on prey responses. We first tested effects on aggregate, community‐level prey responses: (1) total prey abundance (summed across all prey taxa), (2) species richness, (3) Shannon diversity, (4) average prey head width, and (5) the coefficient of variation (CV) of prey head width. These latter two responses were used as measures of prey community size structure. We then considered experimental effects on the abundance of specific taxa—each of the four major insect prey orders separately (Trichoptera [caddisflies], Diptera [true flies], Ephemeroptera [mayflies], Plectoptera [smaller, non‐Perlid stoneflies]), as well as the abundance of each of the four most common prey taxa found in enclosures (family Chironomidae, genus *Dolophilodes* [order Trichoptera], genus *Glossosoma* [order Trichoptera], genus *Hydropsyche* [order Trichoptera]). All responses were measured at the enclosure (*n* = 65) level.

We built LMMs using the lme4 package (Bates et al. 2023) in R version 4.2.0 (R Core Team 2023). All LMMs included the enclosure‐specific current velocity and stream temperature, both measured at the end of each 72‐h block, as additional independent variables. We used final rather than initial near‐bed current velocity because initial current velocity was kept as similar as possible among enclosures within blocks. All LMMs additionally included temporal block (1–6) modeled as a random effect. LMMs were fit using restricted maximum likelihood (REML) and residuals were analyzed using the DHARMa package (Hartig and Lohse 2022). Model fits satisfied LMM assumptions including normality of residuals assessed through visual inspection of quantile‐quantile plots and homogeneity of variance using Levene's test (Schielzeth et al. 2020).

We used the same procedure to test for treatment and environmental effects in all LMMs. First, we built models that included both predator‐present treatments and no‐predator controls (the entire data set), with “treatment” modeled as a factor with five levels ([1] control, [2] small stoneflies, [3] small stoneflies with glued mouthparts, [4] large stoneflies, [5] large stoneflies with glued mouthparts). Initial models also included 2‐way interactions between treatment and each environmental variable (current velocity and stream temperature), but these interactions were never significant and were dropped before proceeding with hypothesis testing.

To test the overall effect of stonefly predators on prey responses irrespective of stonefly size structure and mouthpart gluing, we performed planned linear contrasts between grouped predator‐present treatments (*n* = 48 enclosures) and the no‐predator control (*n* = 17 enclosures) using the “emmeans” package in R (Lenth et al. 2023). Degrees of freedom for linear contrasts were approximated using the Kenward–Roger method. We also used LMMs applied to the entire data set (including controls) to test for independent effects of near‐bed current velocity and stream temperature on prey responses. Significance testing for effects of environmental variables was accomplished using the anova function in the lmerTest package (Kuznetsova et al. 2018) which applies Type III ANOVAs with degrees of freedom approximated using Satterthwaite's method.

Next, we dropped data from control enclosures and built LMMs with stonefly predator size (small versus large) and mouthpart gluing (unglued versus glued) as interactive factors. Environmental variables and the random effect of temporal block were retained in models. A significant interaction between predator size and mouthpart gluing would indicate that the relative importance of NCEs versus CEs depends on stonefly size structure. Again, Type III ANOVAs were applied to models to test effects of stonefly predator size, mouthpart gluing and their interaction. An Akaike Information Criterion model ranking approach yielded the same qualitative results.

We used permutational multivariate analysis of variance (PERMANOVA) to test for experimental and environmental effects on multivariate prey community structure. PERMANOVA was based on Bray‐Curtis distances derived from prey abundance data and carried out using the adonis2 function in the package vegan (Oksanen et al. 2020) in R. We dropped five rare taxa prior to multivariate analysis, each with ≤ 8 observations out of 4406 total observations. We modeled treatment as a factor with five levels and included enclosure‐specific current velocity and stream temperature as additional fixed effects. Dispersions calculated using the betadisper function in the package vegan were homogeneous across treatment groups (ANOVA: *F*_4,60_ = 0.935, *p* = 0.45). To account for the temporal structure of our data, the strata argument in the adonis2 function was used to constrain permutations within temporal blocks. We ran 999 permutations in total. Differences in multivariate community structure were visualized using rank‐based nonmetric multidimensional scaling (nMDS). We compared ordinations using 2 (stress = 0.214, 20 iterations) versus 3 (stress = 0.154, 20 iterations) dimensions, though doing so did not change the patterns we observed. We present the first 2 axes from the 3‐dimensional ordination.

## 3 Results

### 3.1 Environmental Variables

All stoneflies (*n* = 144) survived over the course of the experiment. Stonefly glue masks were always found intact after experimental trials and post hoc stomach dissections revealed empty guts of glued stoneflies. Near‐bed current velocity varied among blocks (ANOVA: *F*_5,59_ = 22.362, *p* < 0.001) but showed no consistent trend over time (mean ± 1 SD = 56.51 cm s^−1^ ± 29.23). Stream temperature increased over the six temporal blocks from 15°C to 21°C.

### 3.2 Predator Effects on Prey Community Responses

**FIGURE 1:**
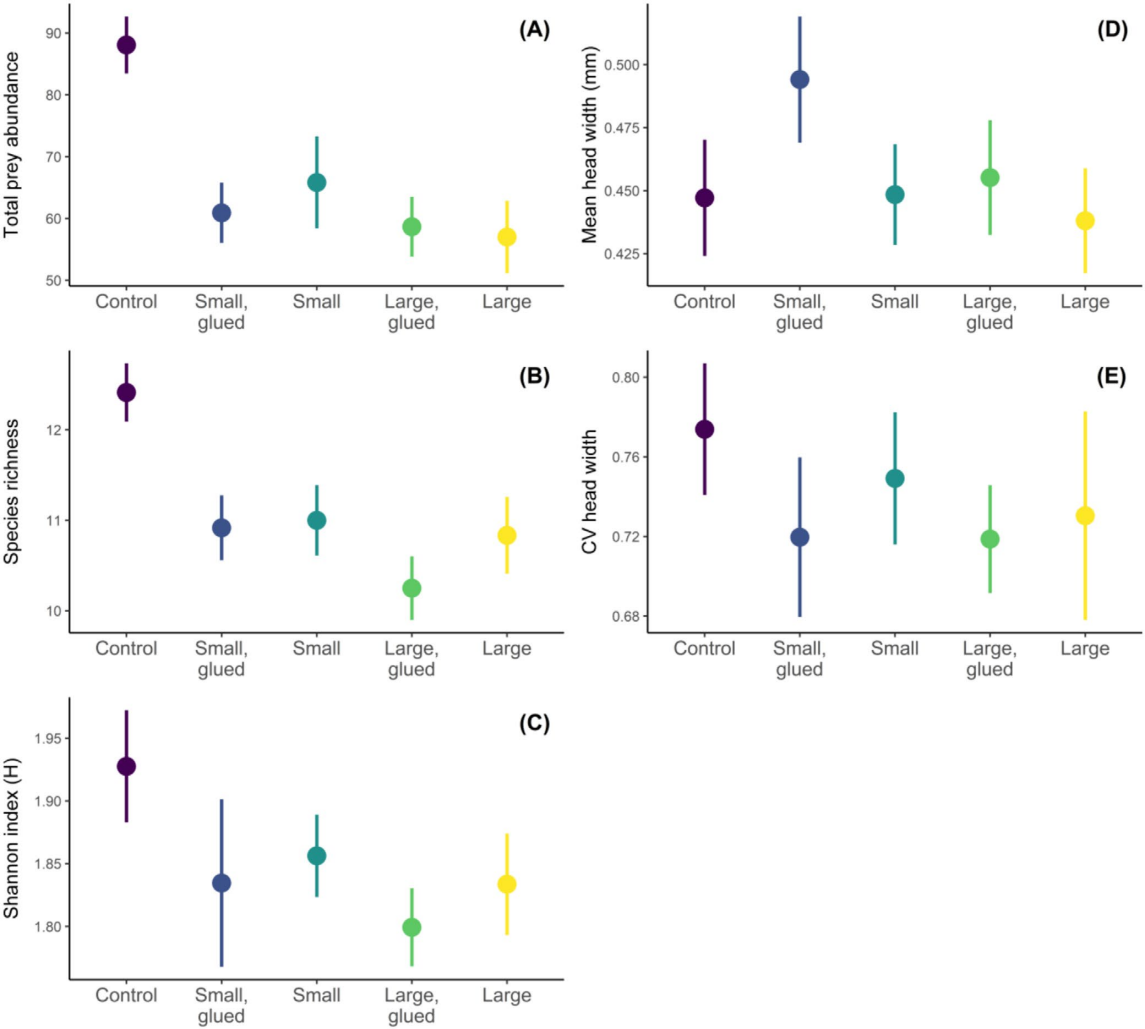
Effects of stonefly predators and manipulations to stonefly size structure and feeding ability on benthic invertebrate community responses. (A) Total prey abundance per enclosure, (B) species richness, (C) Shannon index, (D) mean prey head width, and (E) coefficient of variation (CV) of prey head width. Stonefly size structure was manipulated using two levels, four small stonefly individuals (“small”) versus 2 large stonefly individuals (“large”), and the mouthparts of these stoneflies were either unmanipulated or glued (“glued”). Points represent means and error bars represent ±1 standard error. The control was replicated 17 times, while each treatment was replicated 12 times (*n* = 65 enclosures total).

Stoneflies reduced the total abundance (planned contrast: *t*_1,55.1_ = 5.159, *p* < 0.001; Figure 1A), species richness (planned contrast: *t*_1,55.5_ = 5.765, *p* < 0.001; Figure 1B) and Shannon diversity (planned contrast: *t*_1,55.5_ = 2.681, *p* = 0.01; Figure 1C) of the invertebrate prey community relative to control enclosures. The reduction in total prey abundance was considerable, 32% or ~28 individuals per enclosure, while stonefly effects on prey species richness (14%, ~1.75 species) and Shannon diversity (20%, *H* = 0.106) were less pronounced. Stoneflies did not influence mean prey head width (planned contrast: *t*_1,54.5_ = 0.625, *p* = 0.534; Figure 1D) or the CV of prey head width (planned contrast: *t*_1,54.5_ = 0.907, *p* = 0.368; Figure 1E). Manipulations to stonefly size structure, feeding ability (i.e., mouthpart gluing), and their interaction had no effect on abundance‐based or size‐based prey community responses (ANOVA: *p* ≥ 0.098; Figure 1).

**FIGURE 2:**
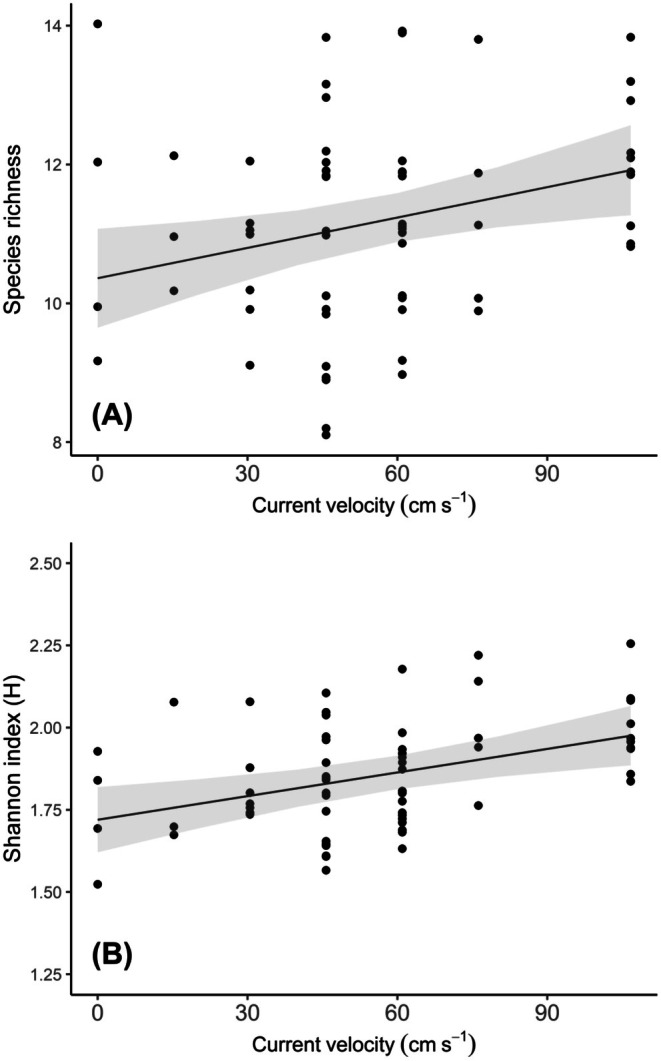
Significant effects of near‐bed current velocity on (A) species richness and (B) species evenness of the benthic invertebrate prey community in enclosures (*n* = 65). Lines and shaded regions depict general linear mixed model fits and 95% confidence intervals, respectively. Vertical jitter was added to data points to avoid overplotting.

**FIGURE 3:**
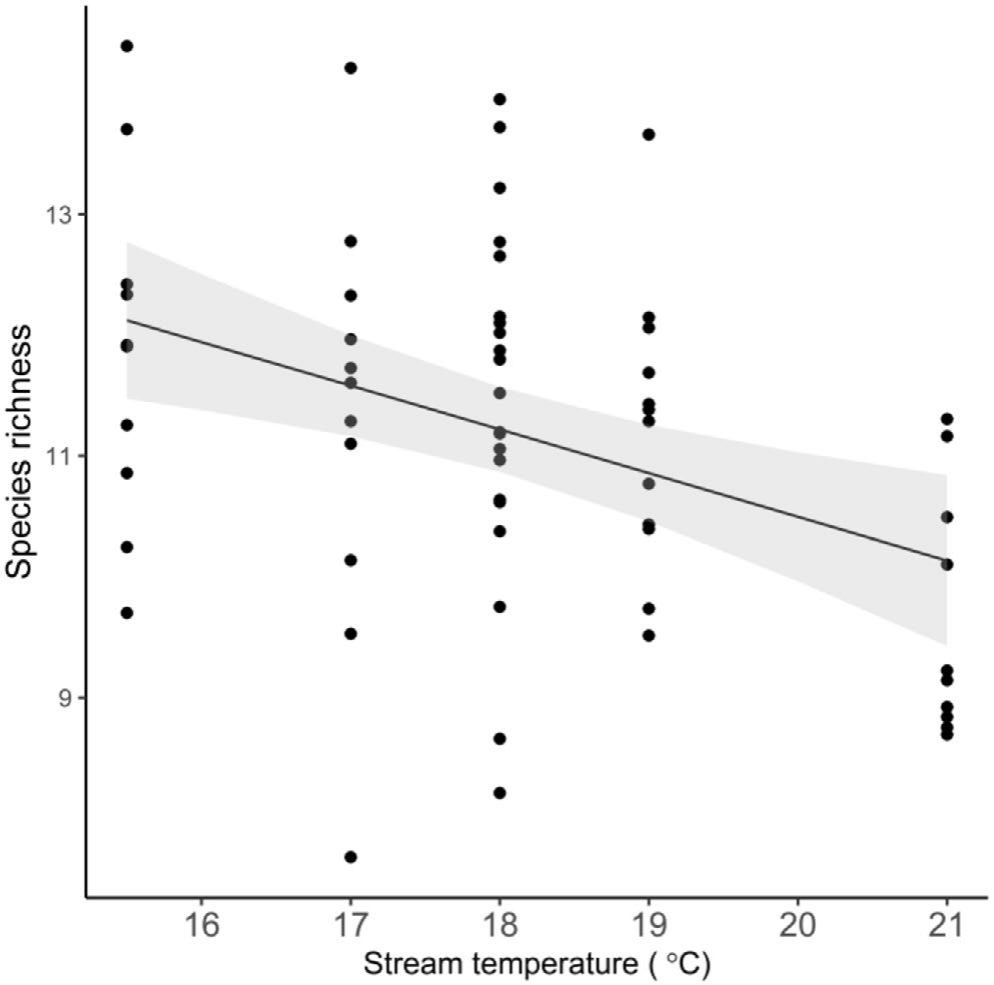
Significant effect of stream temperature on the species richness of the benthic invertebrate prey community in enclosures (*n* = 65). Line and shaded region depict general linear mixed model fits and 95% confidence intervals, respectively. Vertical jitter was added to data points to avoid overplotting.

Enclosures that experienced relatively high current velocity by the end of trials supported greater species richness (ANOVA: *F*_1,12.9_ = 7.143, *p* = 0.019) and evenness (Shannon index) (ANOVA: *F*_1,13.6_ = 13.639, *p* = 0.007) (Figure 2), while increasing stream temperature reduced prey species richness (ANOVA: *F*_1,4.1_ = 11.782, *p* = 0.026; Figure 3). We detected no environmental effects on total prey abundance, mean prey head width or the CV of prey head width (ANOVA: *p* ≥ 0.185).

### 3.3 Predator Effects on Specific Prey Taxa

**FIGURE 4:**
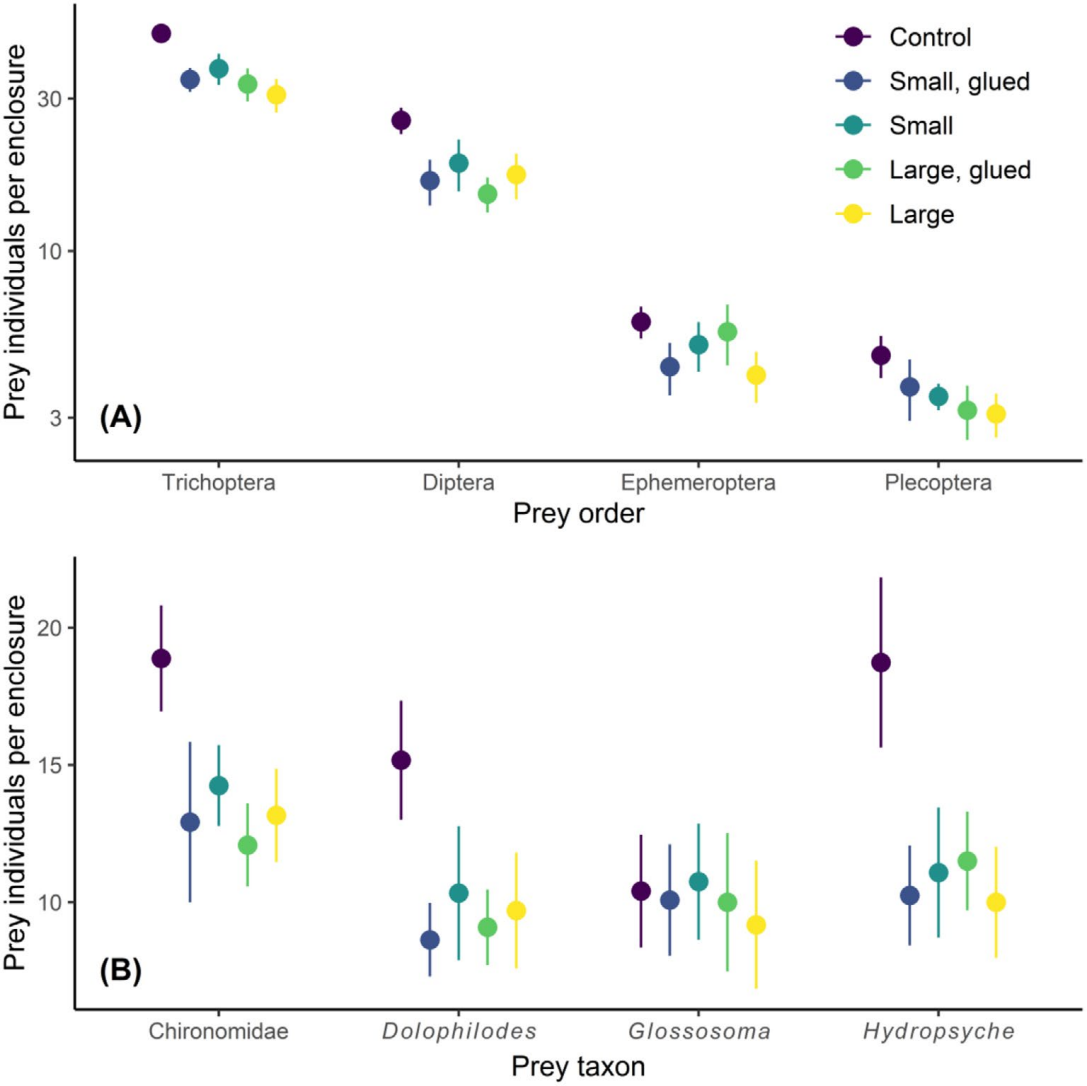
Effects of stonefly predators and manipulations to stonefly size structure and feeding ability on the abundance of (A) the four major insect prey orders (Trichoptera [caddisflies], Diptera [true flies], Ephemeroptera [mayflies], Plecoptera [stoneflies]), and (B) the four most common prey taxa found in enclosures (family Chironomidae [order Diptera], genus *Dolophilodes* [order Trichoptera], genus *Glossosoma* [order Trichoptera], genus *Hydropsyche* [order Trichoptera]). Abundance data in panel A are plotted on a log scale. Stonefly size structure was manipulated using two levels, four small stonefly individuals (“small”) versus two large stonefly individuals (“large”), and the mouthparts of these stoneflies were either unmanipulated or glued (“glued”). Points represent means and error bars represent ±1 standard error. The control was replicated 17 times, while each treatment was replicated 12 times (*n* = 65 enclosures total).

The orders Trichoptera and Diptera dominated enclosure samples (56% and 28% of all samples, respectively), while Ephemeroptera and Plecoptera were relatively rare (8% and 5% of all samples, respectively). The presence of stoneflies in enclosures reduced the abundance of Trichoptera (planned contrast: *t*_1,54.7_ = 4.527, *p* < 0.001), Diptera (planned contrast: *t*_1,54.4_ = 3.371, *p* = 0.001), and Plecoptera (planned contrast: *t*_1,55_ = 2.076, *p* = 0.043), but had no effect on the abundance of Ephemeroptera (planned contrast: *t*_1,55_ = 1.371, *p* = 0.176) (Figure 4A). We detected no environmental effects or additional treatment effects (stonefly size structure, feeding ability, and their interaction) on the abundance of these four insect orders (ANOVA: *p* ≥ 0.145).

**FIGURE 5:**
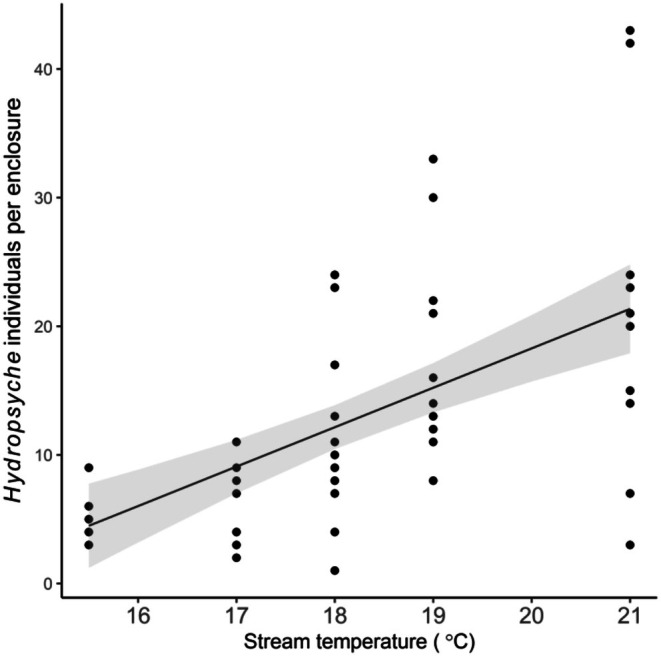
Significant effect of stream temperature on the abundance of the caddis *Hydropsyche* per enclosure (*n* = 65). Line and shaded region depict general linear mixed model fits and 95% confidence intervals, respectively.

The four most common prey taxa found in enclosures included the family Chironomidae (76% of all Dipterans), the genus *Dolophilodes* (28% of all Trichopterans), the genus *Glossosoma* (26% of all Trichopterans) and the genus *Hydropsyche* (33% of all Trichopterans). Stoneflies reduced the abundance of Chironomids (planned contrast: *t*_1,55.8_ = 2.982, *p* = 0.004), *Dolophilodes* (planned contrast: *t*_1,51.3_ = 2.890, *p* = 0.006) and *Hydropsyche* (planned contrast: *t*_1,55.2_ = 3.510, *p* < 0.001), but not the abundance of the cased caddis *Glossosoma* (planned contrast: *t*_1,53.2_ = 1.513, *p* = 0.136) (Figure 4B). Stream temperature increased the abundance of *Hydropsyche* (ANOVA: *F*_1,56_ = 33.949, *p* < 0.001; Figure 5). We detected no additional environmental or treatment effects (stonefly size structure, feeding ability, and their interaction) on the abundance of these four numerically dominant insect taxa (ANOVA: *p* ≥ 0.102).

### 3.4 Predator Effects Multivariate Prey Community Structure

**FIGURE 6:**
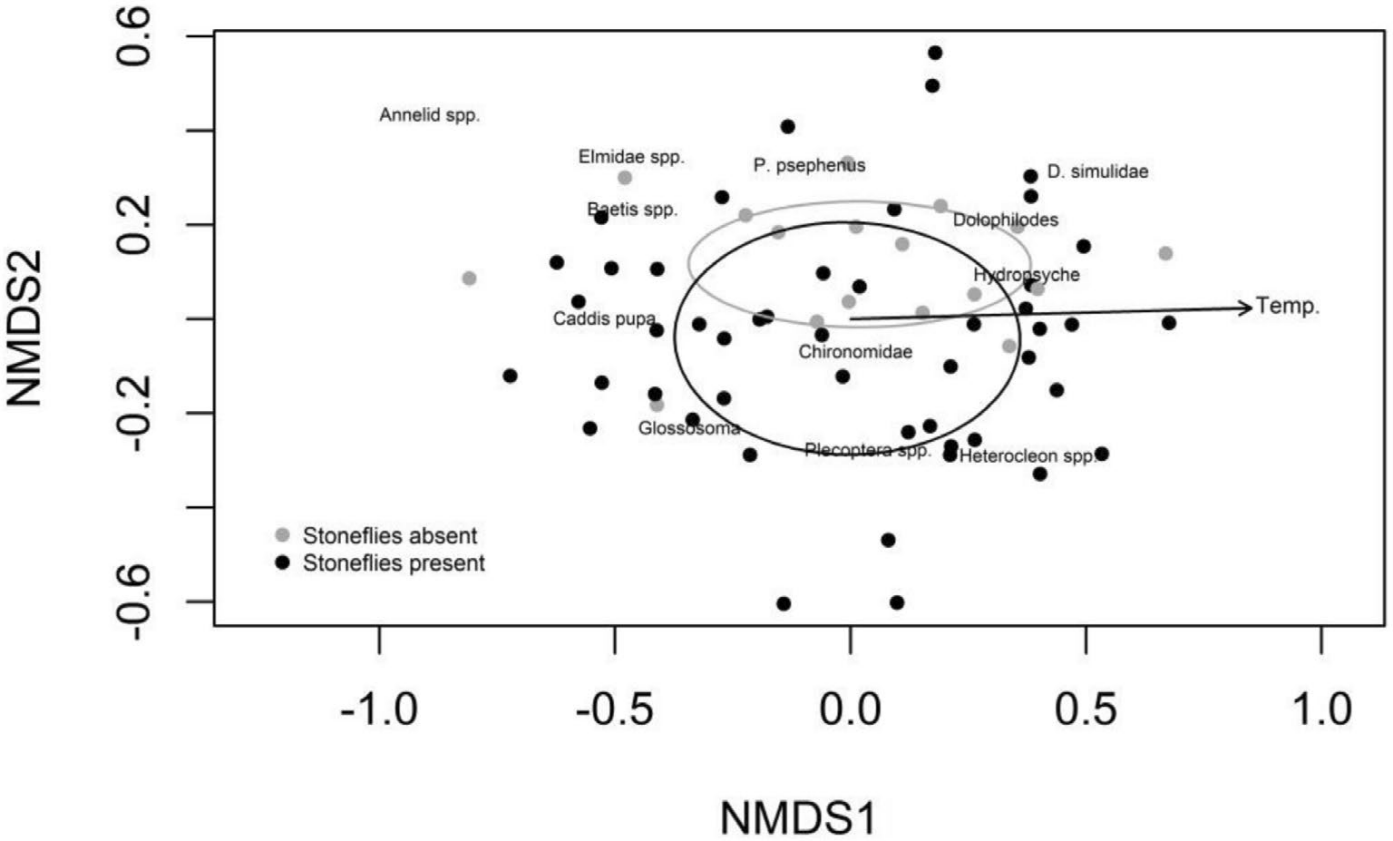
Nonmetric multidimensional scaling (nMDS) visualization of benthic invertebrate community composition (stress = 0.154, 20 iterations). Points represent the composition of communities within enclosures (*n* = 65) based on Bray–Curtis dissimilarities. Gray points are stonefly absent control enclosures while black points are enclosures with stonefly predators added. Ellipses depict 95% confidence intervals surrounding each group. Vector represents the significant correlation between stream temperature (“Temp.”) and community composition.

Multivariate analysis largely supported the results of univariate analyses. The presence of stoneflies altered the structure of the prey community (PERMANOVA: *F*_4,58_ = 1.475, *p* = 0.025), but this predator effect did not further depend on stonefly manipulations—stonefly body size, stonefly mouthpart gluing, and their interaction did not influence community structure (PERMANOVA: *p* > 0.257). The second, vertical NMDS axis (stress = 0.154, 20 iterations; Figure 3) shows that stonefly presence is associated with the cased caddis *Glossosoma*, non‐Perlid stonefly species and the mayfly *Heterocleon* (Family Baetidae), which made up 82% of all mayflies sampled. We observed an additional effect of increasing stream temperature over the course of the experiment on prey community structure as indicated by variation along the first, horizontal NMDS axis (PERMANOVA: *F*_1,58_ = 13.282, *p* = 0.006; Figure 6).

## 4 Discussion

Predator size structure varies over space and time due to natural and anthropogenic causes, but its role in mediating NCEs on prey communities is poorly resolved. Our field experiment tested the role of predator body size as a contextual factor governing the strength of NCEs relative to CEs in a headwater stream food web. Stonefly predators induced strong and pervasive NCEs on prey, but these effects were robust to our alteration of stonefly size structure. Our findings suggest that predator biomass alone may predict the NCEs that shape aquatic insect community structure and distribution in streams.

### 4.1 No Effect of Size Structure on Nonconsumptive Predator Effects

Our experiment was designed to distinguish two mechanisms by which predator size structure can influence NCEs. The first (null) mechanism is that size structure influences NCEs through its effect on predator biomass. Under this mechanism, predator biomass determines the concentration of predator cues in the environment, driving prey behavioral changes (i.e., NCEs) in a dose‐dependent manner (Hill and Weissburg 2013). Alternatively, prey may distinguish predator size independently from biomass. For example, fathead minnows ( *Pimephales promelas* ) exhibited stronger anti‐predator behavioral responses to kairomones from small pike ( *Esox lucius* ) than from large pike, even though chemical cues from large pike were more concentrated (Kusch et al. 2004). Small and large pike were also fed the same diet in this study, ruling out any immediate effects of kairomone differences in driving differences in anti‐predator behavior.

Our finding that small versus large stoneflies with equivalent total biomass caused the same NCEs supports the null biomass effect, though an additional experiment that manipulates stonefly biomass and measures NCEs is necessary to test this mechanism. Predator biomass determination of NCEs might be common in turbid or turbulent aquatic environments where vision is impaired and kairomones play an important role in predator detection. For example, in estuaries, differences in blue crab ( *Callinectes sapidus* ) predator biomass, but not body size, dictate the strength of mud crab ( *Panopeus herbstii* ) prey behavioral responses to blue crab chemical cues (Hill and Weissburg 2013). Likewise, mayflies use kairomones to detect stonefly predators in turbulent stream riffles prior to physical contact (Peckarsky 1980; Peckarsky and Dodson 1980; Peckarsky and Penton 1989). Interestingly, there is evidence that tactile cues play an important role in stonefly detection at closer range, with some studies reporting that physical contact is necessary to induce a behavioral response of prey (Ode and Wissinger 1993; Hoover and Richardson 2010). How tactile cues scale with predator size structure, biomass, and density is less clear and requires experimental attention.

Though ontogenetic niche shifts are not a prerequisite for prey discrimination of predator body size (Kusch et al. 2004), we propose that ontogenetic niche shifts could increase the likelihood of such. Diet and foraging mode differences among predator species are major factors explaining variation in the strength of NCEs (Orrick et al. 2024). Likewise, diet and foraging mode differences within predator species should increase the likelihood that prey discriminate different‐sized predator individuals and exhibit behavioral responses concomitant with the risk they pose. While stoneflies are known to exhibit ontogenetic niche shifts (Tierno de Figueroa and López‐Rodríguez 2019), it is possible that niche shifts did not occur during our study, or aspects of our enclosure experiment limited their occurrence. Preliminary gut contents analysis of stoneflies collected within the time frame of our study revealed similar degrees of carnivory between the two experimental stonefly size classes. Instead, shifts between omnivory and carnivory may emerge seasonally due to temperature‐dependent physiological constraints or changes in resource availability (Miyasaka and Genkai‐Kato 2009). Our enclosure design could have also limited stonefly access to different food resources or constrained foraging behavior, thereby diminishing size‐based differences in predator cues.

### 4.2 Pervasive Nonconsumptive Effects

While NCEs are often considered in the context of pairwise predator–prey interactions, our field experiment measured NCEs across a diverse prey community. We found that stoneflies reduced the abundances of most insect prey taxa and prey size classes, even when incapable of feeding. This effect of stoneflies can be in part explained by prey emigration from enclosures, permitted by the large, 2 mm mesh we used for enclosure paneling (Cooper et al. 1990). Diverse stream insects actively enter the current and drift to a new, downstream patch in response to deteriorating environmental conditions in their current patch (Waters 1972), including the presence of predators (Malmqvist and Sjöström 1987). This anti‐predator behavioral response has been shown to reduce the feeding rates of stream insects (Malmquist 1992; Scrimgeour and Culp 1994; Peckarsky 1996) and depress the nymphal growth rates and adult fecundity of mayflies (Peckarsky et al. 1993).

Despite the general pattern of reduced insect abundance in the presence of stoneflies, we observed two exceptions—abundances of the caddisfly *Glossosoma* and mayflies (Ephemeroptera) were unaffected by stoneflies. Multivariate analysis supported this effect as stonefly present enclosures were characterized by the predominance of these two insect prey groups. *Glossosoma* inhabits a portable case built from pebbles. This constitutive defense mechanism limits mobility and reduces the need for behavioral responses to stonefly predation risk (Kohler and McPeek 1989; Ruetz et al. 2004). Lack of stonefly effects on mayfly abundance is more difficult to explain. The mayflies that dominated our samples (Family Baetidae) are sometimes selected by *Acroneuria abnormis* (Genito and Kerans 1999) and Perlid stoneflies have been shown to depress their individual growth and abundance in experiments (Peckarsky and Penton 1989; Peckarsky et al. 1990, 1993). However, the relative abundance of mayflies was low in our study in part due to the late summer period, which limited our statistical power to detect an effect. Regardless of the mechanism, both these taxa are important grazers of algae biofilms on rock surfaces, and resistance to stonefly NCEs could disrupt a trophic cascade on primary production (Wellnitz 2014).

A related finding of our study was that stoneflies with glued mouthparts reduced insect prey abundance to the same degree as unmanipulated stoneflies. While this finding suggests that stonefly CEs are less important than NCEs in determining prey community structure, our experiment lacked a treatment that isolated CEs and thus provided a more rigorous test of relative importance. Furthermore, CEs did occur—the stomachs of unmanipulated stoneflies occasionally contained insect prey (5/72 unmanipulated stoneflies), while glued stonefly stomachs were always empty. While the rarity of stomach contents of unmanipulated stoneflies might suggest weak CEs, we attribute this rather to the timing of stonefly sampling. Stoneflies feed nocturnally and had digested most stomach contents by our midday sampling period.

Our results can be compared to other experiments that manipulated stoneflies in streams to measure predatory impacts. Early studies (Peckarsky and Dodson 1980; Peckarsky 1985) measured stonefly effects on prey colonization into enclosures, rather than stocking enclosures with prey as in our study. To isolate noncontact predator cues, these studies restrained stoneflies within smaller cages inside enclosures. In contrast, more recent studies (Wellnitz 2014, the present study) glued stonefly mouthparts, preserving tactile cues and more accurately isolating NCEs. Despite these differences in approach, all of these studies demonstrate a major role for NCEs in making up total stonefly impacts on prey in streams.

### 4.3 Environmental Effects on Invertebrate Communities

In addition to predator and prey traits, aspects of the physical environment can modify the strength of NCEs (Wirsing et al. 2021). Within streams, near‐bed current velocity has been shown to mediate the top‐down effects of stoneflies on insect prey (Peckarsky et al. 1990) with cascading effects on algae resource abundance (Wellnitz 2014). For example, Peckarsky et al. (1990) show that the stonefly *Dinocras cephalotes* only reduces prey abundance in locations with high current velocity, which this stonefly species also selects for habitat. Wellnitz (2014) found that stoneflies promote benthic algae biomass via a behaviorally mediated trophic cascade (indirect NCE), but only under slow current velocity. While we tested for interactions between predator treatments and current velocity, these effects were never significant and thus were dropped early in our modeling procedure. Our failure to detect a mediating effect of current velocity could reflect stonefly species‐level differences in current preferences (Peckarsky et al. 1990), or that effects only emerge across a wider range of current velocities.

Instead, environmental conditions, current velocity and stream temperature, influenced the invertebrate prey community independently from stonefly predators. Current velocity had positive effects on invertebrate diversity, increasing both species richness and evenness in enclosures. This effect may be due in part to increased colonization rates of enclosures in relatively high current velocity locations over the course of the experiment, or that certain species require faster current velocities for efficient feeding (Degani et al. 1993). In contrast, increasing stream temperature reduced species evenness, a commonly reported effect of warming on stream macroinvertebrate communities (Bonacina et al. 2023). Importantly, the high temperatures (~21°C) observed near the end of our experiment are near peak temperatures for Tankerhoosen Brook and likely intolerable by stenothermal species (Dietrich et al. 2023). In support of this mechanism, increasing stream temperature over the course of our experiment increased the abundance of the caddis *Hydropsyche*, a habitat generalist, eurythermal species that can tolerate a broad range of environmental conditions.

## 5 Conclusion

Recent work advocates trait‐based approaches to explaining variation in the strength of NCEs within and across ecosystems (Wirsing et al. 2021). In short, the traits of predators (e.g., hunting mode, space use) might determine their detectability by prey, and predators that are more detectable should induce stronger prey behavioral changes (i.e., NCEs). While studies thus far have focused on trait differences among predator species, we lack understanding of whether prey respond behaviorally to widespread phenotypic variation within predator species. Though our study supports similar prey behavioral responses to different predator size classes, further research should examine effects of a wider range of predator traits known to vary over ontogeny (Werner and Gilliam 1984) and consider effects on prey species that rely on nonchemical modes of predator detection (Wirsing et al. 2021). Given widespread anthropogenic modifications to trait distributions within predator populations, this work will help clarify changes to the top‐down control of ecosystems.

## Author Contributions

**Benjamin J. Toscano:** conceptualization (lead), formal analysis (lead), investigation (equal), writing – original draft (lead), writing – review and editing (equal). **Alyce Segal:** conceptualization (supporting), investigation (equal), writing – original draft (supporting), writing – review and editing (equal). **Martina Exnerova:** investigation (equal), writing – review and editing (equal). **Mia A. Ver Pault:** investigation (equal), writing – review and editing (equal).

## Ethics Statement

Organisms were collected under the permission of the Connecticut Department of Energy and Environmental Protection (Permit SC‐22006).

## Conflicts of Interest

The authors declare no conflicts of interest.

## Data Availability

The data set is deposited in the Dryad repository https://doi.org/10.5061/dryad.905qfttzs.
